# Endoplasmic Reticulum Stress Caused by Lipoprotein Accumulation Suppresses Immunity against Bacterial Pathogens and Contributes to Immunosenescence

**DOI:** 10.1128/mBio.00778-17

**Published:** 2017-05-30

**Authors:** Jogender Singh, Alejandro Aballay

**Affiliations:** Department of Molecular Genetics and Microbiology, Duke University Medical Center, Durham, North Carolina, USA; Massachusetts General Hospital

**Keywords:** *Caenorhabditis elegans*, ER stress, *Pseudomonas aeruginosa*, immune senescence, innate immunity, lipoproteins, unfolded protein response

## Abstract

The unfolded protein response (UPR) is a stress response pathway that is activated upon increased unfolded and/or misfolded proteins in the endoplasmic reticulum (ER), and enhanced ER stress response prolongs life span and improves immunity. However, the mechanism by which ER stress affects immunity remains poorly understood. Using the nematode *Caenorhabditis elegans*, we show that mutations in the lipoproteins vitellogenins, which are homologs of human apolipoprotein B-100, resulted in upregulation of the UPR. Lipoprotein accumulation in the intestine adversely affects the immune response and the life span of the organism, suggesting that it could be a contributing factor to immunosenescence. We show that lipoprotein accumulation inhibited the expression of several immune genes encoding proteins secreted by the intestinal cells in an IRE-1-independent manner. Our studies provide a mechanistic explanation for adverse effects caused by protein aggregation and ER stress on immunity and highlight the role of an IRE-1-independent pathway in the suppression of the expression of genes encoding secreted proteins.

## IMPORTANCE

Increased accumulation of unfolded and/or misfolded proteins in the endoplasmic reticulum (ER) leads to enhanced ER stress. However, the mechanism(s) by which ER stress affects immunity remain understudied. Using the nematode *C. elegans*, we showed that mutations in lipoproteins lead to their accumulation in the intestine, causing ER stress and adversely affecting the life span of the organisms and their resistance to pathogen infection. Our results indicate that the ER stress caused by lipoprotein accumulation significantly reduced the levels of expression of genes encoding secreted immune effectors, contributing to immunosenescence. It is known that ER stress may suppress gene expression via IRE-1, which is a sensor of ER stress. The novel mechanism uncovered in our study is IRE-1 independent, which highlights the role of a novel process by which ER stress suppresses innate immunity.

## INTRODUCTION

The endoplasmic reticulum (ER) is a principal site where newly synthesized transmembrane and secretory proteins are folded, assembled, and undergo maturation. An increased accumulation of misfolded proteins in the ER can lead to ER stress (1). Indeed, a number of naturally occurring mutations detected in genetic diseases lead to aberrant protein folding, resulting in the ER stress observed in human diseases, including neurodegenerative diseases, diabetes, cancer, and inflammatory diseases (2–5). To cope with the ER stress, a series of unfolded protein response (UPR) pathways are activated. The UPR is an integrated intracellular signaling pathway that links the ER lumen to the cytoplasm and nucleus. Three parallel branches of the UPR—*ire-1/xbp-1*, *atf-6*, and *perk-1*—help to maintain ER homeostasis via a number of sophisticated mechanisms that control translation, protein degradation, mRNA degradation, and chaperone levels (6, 7). However, the UPR becomes compromised with age (8, 9), leading to enhanced protein aggregation in the ER.

Recent studies have shown that the UPR plays crucial roles in the regulation of the life span and immunity of organisms (9–11). Constitutive expression of the active form of XBP-1, that is part of the most conserved branch of the UPR, has been found to prolong life span (9). It has also been shown that bacterial infection causes damage to proteins at the site of infection, which leads to their aggregation (12), and that bacterial infection upregulates the UPR (13, 14). In addition, an increase in the UPR enhances the immunity of animals against bacterial infection (15–17).

We conducted a forward genetic screen in the nematode model organism, *Caenorhabditis elegans*, to identify dominant mutants exhibiting high UPR levels. We obtained several mutant animals with an upregulated UPR. Whole-genome sequencing (WGS) revealed that the UPR upregulation in these mutants resulted from mutations in vitellogenin proteins (VIT), which are lipoproteins involved in the transport of lipids from the intestine to oocytes and are homologs of human apolipoprotein B-100 (apoB-100). We show that mutations in lipoproteins result in their accumulation in the intestine, which causes ER stress and adversely affects the life span of the organisms and their resistance to pathogen infection. The upregulated UPR counteracts the toxic effects of ER stress and improves immunity and prolongs the life span of the mutants. Our studies also suggest that accumulation of lipoproteins may be a factor in immunosenescence. Quantification of the levels of expression of genes using RNA sequencing showed that ER stress caused by lipoprotein accumulation significantly reduced the expression levels of genes encoding secreted immune effectors. Our studies uncovered a novel mechanism by which ER stress suppresses innate immunity.

## RESULTS

### Forward genetic screen for mutants exhibiting UPR upregulation.

**FIG 1:**
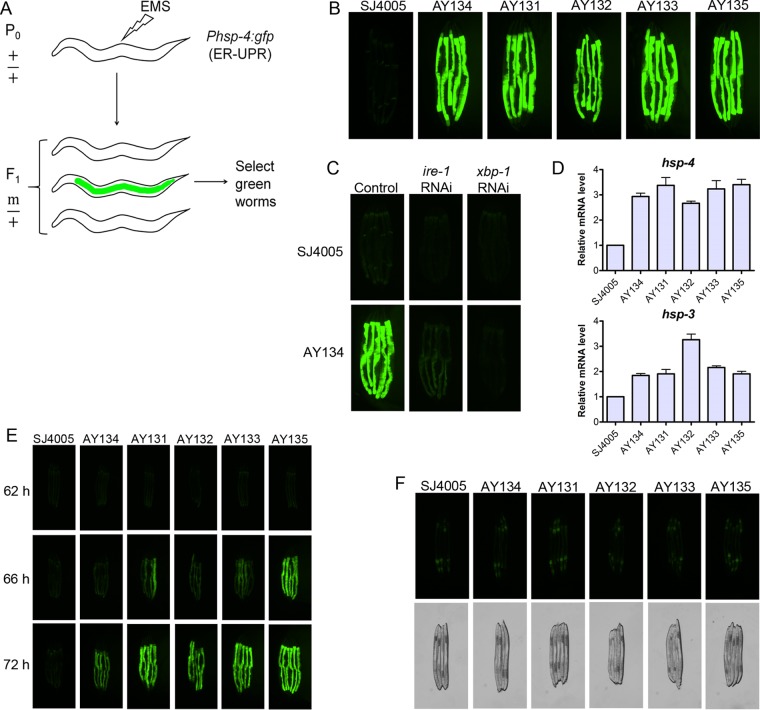
Forward genetic screen for mutants exhibiting an upregulated UPR. (A) Schematic for the forward genetic screen. The SJ4005 strain, which expresses *Phsp-4*::*gfp* and is a reporter strain for the ER UPR, was used for the screen. Mutants with high expression levels of GFP were screened from the F_1_ progeny after mutagenesis. (B) Fluorescence images of five of the mutant animals and SJ4005 animals containing the reporter *Phsp-4*::*gfp*. The mutants isolated in the screen were designated AY131 to AY135. (C) Fluorescence images of one of the mutants and SJ4005 animals following RNAi against the UPR genes *ire*-*1* and *xbp-1*. Animals grown on empty RNAi vector were used as the control. (D) Quantitative reverse transcription-PCR (qRT-PCR) for the ER chaperone genes *hsp-4* and *hsp-3* in the mutant and SJ4005 animals. The bar graphs show the means plus standard deviations (SD) (error bars) from three independent experiments. The *P* values for *hsp-4* RNA levels in mutant strains relative to SJ4005 animals are shown as follows: *P* < 0.001 for AY134, *P* < 0.001 for AY131, *P* < 0.01 for AY132, *P* < 0.001 for AY133, and *P* < 0.001 for AY135. The *P* values for *hsp-3* RNA levels in mutant strains relative to the levels in SJ4005 animals are as follows: *P* < 0.01 for AY134, *P* < 0.01 for AY131, *P* < 0.001 for AY132, *P* < 0.001 for AY133, and *P* < 0.001 for AY135. (E) Fluorescence images of the mutants and SJ4005 animals carrying the reporter *Phsp-4*::*gfp* during development at 20°C. The time represents the time of development from eggs at 20°C on *E. coli* OP50. Eggs represent the time point 0 h (F) Fluorescence images and the corresponding bright-field images of adult males of mutant and SJ4005 animals carrying the reporter *Phsp-4*::*gfp*.

We conducted a forward genetic screen using the UPR reporter strain SJ4005 *Phsp-4*::*gfp* to isolate mutants exhibiting an upregulated UPR (Fig. 1A). The transcription of *hsp-4*, the *C. elegans* ortholog of the mammalian ER-localized Hsp70 chaperone BiP, is upregulated in response to ER stress. An F_1_ screen of approximately 80,000 ethyl methanesulfonate (EMS)-mutagenized haploid genomes resulted in the isolation of seven dominant mutants with enhanced green fluorescent protein (GFP) expression compared to the parental strain SJ4005 (see Fig. S1A in the supplemental material). All of the mutants showed very high levels of GFP in the intestine compared to the SJ4005 animals. We randomly selected five mutants for in-depth analysis (Fig. 1B). The enhanced GFP expression was completely blocked by knockdown of the UPR gene *xbp-1* and partially blocked by knockdown of the UPR gene *ire-1* by RNA interference (RNAi) (Fig.  1C and Fig. S1A). We confirmed activation of the UPR by monitoring the transcript levels of the ER chaperone genes *hsp-4* and *hsp-3* (Fig. 1D). The mutants had significantly higher levels of the ER chaperone transcripts than the SJ4005 animals did. However, the mutants did not show any significant changes in the transcript levels of *hsp-6* (Fig. S1B), a mitochondrial chaperone that is activated in the mitochondrial UPR. The mutants showed a slight but nonsignificant increase in the transcript levels of the cytosolic chaperone *hsp-16.2* (Fig. S1B), but these changes are marginal compared with those in *hsp-16.2* transcript levels upon heat stress (18), which induces *hsp-16.2*.

10.1128/mBio.00778-17.1 FIG S1  Characterization of the mutants with upregulated UPR. (A) Fluorescent images of the mutant animals along with parental strain SJ4005 upon RNAi against *ire*-*1* and *xbp-1* genes. (B) qRT-PCR for the mitochondrial chaperone gene *hsp-6* as well as cytoplasmic heat shock protein *hsp-16.2* in the mutant worms along with parental strain SJ4005. Bar graphs show mean ± SD (error bars) from three independent experiments. The *P* values for *hsp-6* RNA from mutant animals compared to the values for SJ4005 animals are as follows: *P* > 0.05 for AY134, *P* > 0.05 for AY131, *P* > 0.05 for AY132, *P* > 0.05 for AY133, and *P* > 0.05 for AY135. The *P* values for *hsp-16.2* RNA from mutant animals relative to the values from SJ4005 animals are as follows: *P* > 0.05 for AY134, *P* > 0.05 for AY131, *P* > 0.05 for AY132, *P* > 0.05 for AY133, and *P* > 0.05 for AY135. Download FIG S1, TIF file, 1.1 MB. Copyright © 2017 Singh and Aballay. 2017 Singh and Aballay This content is distributed under the terms of the Creative Commons Attribution 4.0 International license.

Interestingly, the mutants did not show enhanced GFP levels in comparison to SJ4005 animals until 62 h of growth at 20°C (Fig. 1E). The levels of GFP started to increase in the mutant animals only at 66 h, which coincides with the developmental stage at which L4 larvae molt into young adults. This finding indicated that the UPR was activated in the mutants only in the adult stage and not in the larval stages. Furthermore, the male mutant animals did not display any upregulation of the UPR compared to the SJ4005 animals (Fig. 1F). Taken together, these observations indicated that activation of the UPR in the mutant animals was specific to the adult hermaphrodites.

### Dominant mutations in vitellogenin/lipoprotein-encoding genes upregulate the UPR.

**FIG 2:**
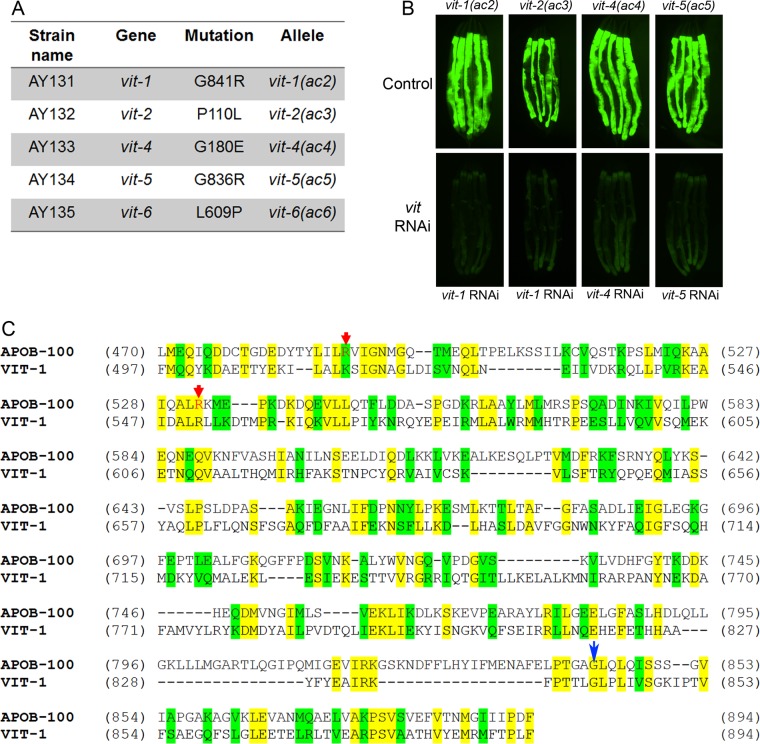
Mutations in vitellogenin genes activate the UPR in the mutants. (A) Table summarizing the mutations in the different *vit* genes identified by whole-genome sequencing in different UPR mutants. The corresponding amino acid changes in the VIT proteins are also shown. (B) Fluorescence images of the UPR mutants containing the reporter *Phsp-4*::*gfp* upon RNAi against *vit* genes. The control is empty vector control RNAi. (C) Sequence alignment of the conserved region of human apolipoprotein B-100 (APOB-100) with *C. elegans* VIT-1. Identical residues (yellow) and similar residues (green) are indicated. Red arrows point at residues whose mutations cause hampered secretion of apoB-100 and are linked with familial hypobetalipoproteinemia. The blue arrow highlights the conserved residue mutated in *vit-1*(*ac2*) and *vit-5*(*ac5*).

To identify the causative mutations, we conducted whole-genome sequencing of the five selected mutants (Fig. 1B). The mutants were backcrossed six times with the parental strain SJ4005, and the sequenced genomes were aligned with the reference genome of *C. elegans*. After subtraction of the common variants, linkage maps of single nucleotide polymorphisms (SNPs) were obtained (Fig. S2). Analysis of the protein-coding genes carrying mutations in the mapped regions of each mutant revealed mutations in one of the six vitellogenin (*vit*) genes present in the *C. elegans* genome (Fig. 2A). VIT proteins are lipoproteins that are expressed in the intestine of the adult hermaphrodite and are involved in the transport of lipids from the intestine to oocytes (19). Moreover, they have a high propensity to misfold and cause ER stress, and they are one of the most abundant proteins in *C. elegans* (20). This high abundance and propensity to cause ER stress may explain why all the mutations corresponded to *vit* genes.

10.1128/mBio.00778-17.2 FIG S2  Mapping of the mutations by whole-genome sequencing. The frequencies of SNPs were plotted against the positions of different chromosomes or linkage groups (LG). The high frequency of SNPs on a particular chromosome compared to other regions of the genome represents the region linked with the causative mutation(s). All mutants except AY135 showed a high frequency of SNPs on chromosome X, while AY135 showed a high frequency of SNPs on chromosome IV. Download FIG S2, TIF file, 0.8 MB. Copyright © 2017 Singh and Aballay. 2017 Singh and Aballay This content is distributed under the terms of the Creative Commons Attribution 4.0 International license.

Consistent with our results indicating that activation of the UPR was observed only in mutant hermaphrodites, *vit* genes are not expressed in males. Moreover, the UPR was upregulated in the mutants only in the intestine (Fig. 1B and Fig. S1A), the site of VIT protein synthesis. We reasoned that if upregulation of the UPR is caused by mutations in *vit* genes, inhibition of the mutated genes should rescue the phenotype. Among the genes encoding vitellogenins, a high level of sequence similarity is observed, resulting in their classification into three groups. The *vit-1* and *vit-2* genes correspond to a group closely related to the group comprising *vit-3*, *vit-4*, and *vit-5*, while *vit-6* has a relatively low similarity to other *vit* genes (Fig. S3). The level of sequence similarity indicates that *vit-1* and *vit-2*, *vit-3*, *vit-4*, and *vit-5* would undergo complete cross RNAi (21). RNAi clones for *vit-2* and *vit-6* are unavailable in the two commercial *C. elegans* RNAi libraries, but given the aforementioned sequence similarity, RNAi against *vit-1* is expected to inhibit *vit-2*. Consistently, RNAi against *vit* genes caused a complete suppression of the enhanced UPR of the tested mutants (Fig. 2B).

10.1128/mBio.00778-17.3 FIG S3  Phylogenetic tree of *vit* genes in *C. elegans*. The phylogenetic tree for the *vit* genes was generated using the web program http://www.phylogeny.fr/. Download FIG S3, TIF file, 0.1 MB. Copyright © 2017 Singh and Aballay. 2017 Singh and Aballay This content is distributed under the terms of the Creative Commons Attribution 4.0 International license.

### Mutations in VIT proteins affect their transport and lead to accumulation in the intestine.

Next, we sought to identify the mechanism by which the dominant mutations in *vit* genes activate the UPR. A previous study showed that reduction of unsaturated fatty acids in membranes upregulates the UPR in *C. elegans* (22). Because VIT proteins are involved in the transport of lipids, we decided to study whether the identified mutations may disturb lipid homeostasis in the intestine. To test whether the UPR upregulation was due to a reduction of unsaturated fatty acids, we supplemented the animal diets with different unsaturated fatty acids. Supplementation of the diet with fatty acids did not suppress the upregulated UPR (Fig. S4), indicating that the UPR was not upregulated due to a reduction of unsaturated fatty acids.

10.1128/mBio.00778-17.4 FIG S4  The enhanced UPR in the mutants remains unaltered upon dietary supplementation of different fatty acids. Fluorescence images of mutant animals carrying the reporter *Phsp-4*::*gfp* grown on *E. coli* OP50 plates containing 300 µM concentration of the corresponding fatty acid. OA, oleic acid; AA, arachidonic acid; EPA, eicosapentaenoic acid. Download FIG S4, TIF file, 1.7 MB. Copyright © 2017 Singh and Aballay. 2017 Singh and Aballay This content is distributed under the terms of the Creative Commons Attribution 4.0 International license.

All of the mutations identified in the VIT proteins are nonconservative in nature. For example, in three isolated mutants, the smallest and neutral amino acid, glycine, is substituted by large and charged amino acids (Fig. 2A). These kinds of amino acid changes may lead to perturbations in the conformation of the protein, potentially resulting in aggregation. Two of the mutants also exhibited the same residue change, glycine for arginine, in a highly conserved region that is also present in human apoB-100 (23) (Fig. 2A and C). This conserved region of apoB-100 is known to have several disease-linked mutations (Fig. 2C), which promote misfolding and hampered secretion (24–26). VIT proteins are expressed in the intestine, from where they are secreted and transported to oocytes so that they can be incorporated into the developing embryos to provide nutrients (19). Thus, we tested whether the upregulated UPR observed in the isolated mutants might be caused by hampered secretion and subsequent accumulation of VIT proteins in the intestine. To examine this possibility, we generated transgenic animals expressing VIT-2::GFP and VIT-2(G839R)::GFP. VIT-2(G839R) carries a mutation analogous to the G841R and G836R mutations found in the *vit-1*(*ac2*) and *vit-5*(*ac5*) mutants.

**FIG 3:**
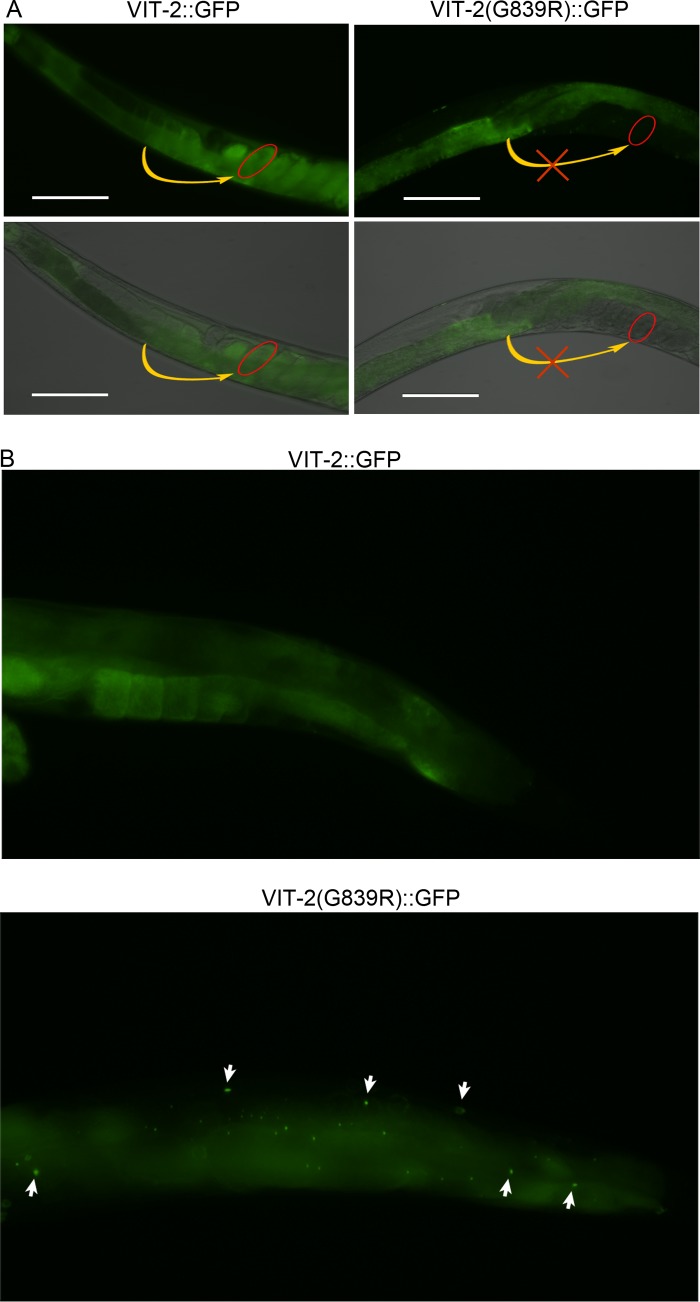
Mutations in VIT proteins lead to their accumulation in the intestine. (A) Fluorescence images of transgenic animals expressing VIT-2::GFP and VIT-2(G839R)::GFP showing that VIT proteins are present in the embryos of VIT-2::GFP-expressing animals but not in the embryos of VIT-2(G839R)::GFP-expressing animals. The red eclipse in the bottom panels are drawn around a single embryo. The yellow curved arrows indicate that the VIT-2 protein is transported to oocytes in VIT-2::GFP-expressing animals but not in VIT-2(G839R)::GFP-expressing animals. The images at the bottom of each column are a merge of the fluorescence and bright-field images. Bars, 100 µm. (B) VIT-2(G839R)::GFP-expressing animals showing the presence of GFP puncta in the intestine as well as the pseudocoelomic region; the VIT-2::GFP-expressing animals do not show any GFP puncta. The white arrows indicate GFP puncta. In all panels, the animals are young adults grown on *E. coli* OP50 at 20°C.

Consistent with earlier studies (19), we observed VIT-2::GFP in both the intestine and embryo (Fig. 3A and Fig. S5). In contrast to VIT-2::GFP, VIT-2(G839R)::GFP remained in the intestine and was not transported to embryos (Fig. 3A). This finding indicated that the G839R mutation could lead to the misfolding and aggregation of VIT proteins, affecting secretion and transport to oocytes. Consistently, VIT-2(G839R)::GFP aggregates were observed in the intestine as well as in the pseudocoelomic region (Fig. 3B). Taken together, these data showed that activation of the UPR in the isolated mutants was caused by the accumulation of VIT proteins in the intestine.

10.1128/mBio.00778-17.5 FIG S5  Mutations in VIT proteins lead to their aggregation. Fluorescence images of transgenic animals expressing VIT-2::GFP and VIT-2(G839R)::GFP. The images at the bottom of each column are merged images of the fluorescence and bright-field images. The arrow in animals expressing VIT-2(G839R)::GFP points toward GFP puncta. The red eclipses in the bottom panels are drawn around a single egg. In both cases, the animals were grown at 20°C on *E. coli* OP50 until young adult stage. Download FIG S5, TIF file, 1 MB. Copyright © 2017 Singh and Aballay. 2017 Singh and Aballay This content is distributed under the terms of the Creative Commons Attribution 4.0 International license.

### Accumulation of VIT proteins leads to enhanced susceptibility to pathogens and a shortened life span.

**FIG 4:**
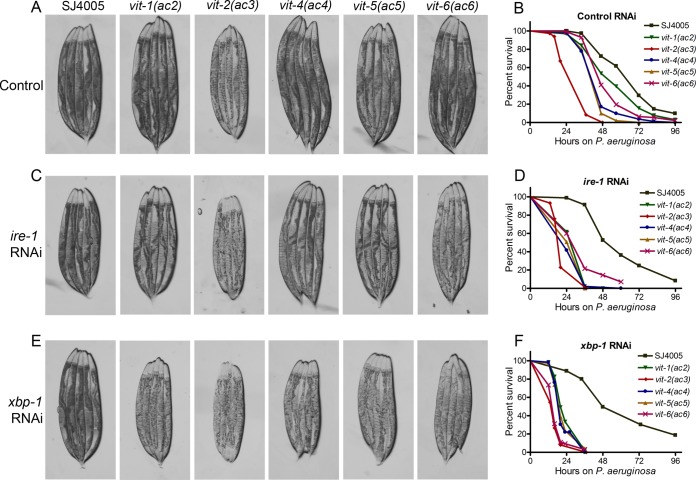
Accumulation of VIT proteins leads to intestinal degeneration. (A) Photomicrographs of the parental strain SJ4005 and mutant animals grown on *E. coli* HT115 containing empty vector at 20°C until they were 1-day-old adults before images were acquired. (B) Survival of SJ4005 and mutant animals on *P. aeruginosa* PA14 at 25°C after treatment with control RNAi. The animals were grown on *E. coli* HT115 containing empty vector at 20°C until they were 1-day-old adults before transferring to *P. aeruginosa* PA14 at 25°C. *P* values comparing the values for mutant animals to the values for SJ4005 animals are as follows: *P* < 0.001 for *vit-1*(*ac2*), *P* < 0.001 for *vit-2*(*ac3*), *P* < 0.001 for *vit-4*(*ac4*), *P* < 0.001 for *vit-5*(*ac5*), and *P* < 0.001 for *vit-6*(*ac6*). (C) Photomicrographs of SJ4005 and mutant animals grown on *E. coli* HT115 containing vector for RNAi against *ire-1* at 20°C until they were 1-day-old adults before images were acquired. (D) Survival of SJ4005 and mutant animals on *P. aeruginosa* PA14 at 25°C after treatment with *ire-1* RNAi. The animals were grown on *E. coli* HT115 containing vector for RNAi against *ire-1* at 20°C until they were 1-day-old adults before being transferred to *P. aeruginosa* PA14 at 25°C. The *P* values for all mutant animals relative to the values for SJ4005 animals are *P* < 0.001. (E) Photomicrographs of SJ4005 and mutant animals grown on *E. coli* HT115 containing vector for RNAi against *xbp-1* at 20°C until they were 1-day-old adults before images were acquired. (F) Survival of SJ4005 and mutant animals on *P. aeruginosa* PA14 at 25°C after *xbp-1* RNAi. The animals were grown on *E. coli* HT115 containing vector for RNAi against *xbp-1* at 20°C until they were 1-day-old adults before being transferred to *P. aeruginosa* PA14 at 25°C. *P* values for all mutant animals relative to SJ4005 animals are *P* < 0.001. Survival curves are representative assays for three independent experiments. *n* = 60 to 100.

On the basis of the previous results, we hypothesized that the accumulation of VIT proteins might affect the intestinal immune response of all isolated mutants. Concomitant blockage of the ATF-6 and XBP-1 branches of the UPR and exposure to bacterial toxins are known to cause intestinal degeneration that results in smaller animals and paler intestines that are visible under the light microscope (13, 27). As shown in Fig. 4A, *vit-2*(*ac3*) animals exhibited smaller sizes and paler appearances compared to the parental strain SJ4005, and these morphological changes correlated with a strong susceptibility to infection by the pathogenic bacterium *Pseudomonas aeruginosa* PA14 (Fig. 4B and Table S1).

10.1128/mBio.00778-17.9 TABLE S1  Summary of survival curves obtained under different conditions Download TABLE S1, XLSX file, 0.01 MB. Copyright © 2017 Singh and Aballay. 2017 Singh and Aballay This content is distributed under the terms of the Creative Commons Attribution 4.0 International license.

When the UPR was downregulated by RNAi against *ire-1*, *vit-2*(*ac3*) and *vit-6*(*ac6*) animals showed strong morphological changes associated with degeneration (Fig. 4C), while RNAi against *xbp-1* imposed those changes in all *vit* mutants (Fig. 4E). In contrast with the results shown in Fig. 4B, all of the mutants showed a strong susceptibility to pathogen infection when RNAi against *ire-1* was used (Fig. 4D and Table S1). The susceptibility to pathogen infection was even stronger in mutants where *xbp-1* was inhibited by RNAi (comparison of Fig. 4D and F). Consistent with these results, *xbp-1* RNAi inhibited the observed UPR more efficiently than *ire-1* RNAi did (Fig. S1A).

**FIG 5:**
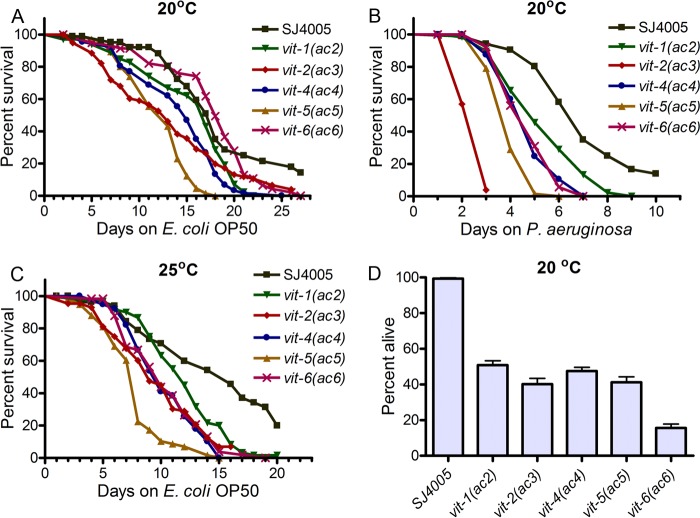
VIT protein accumulation adversely affects life span and enhances pathogen susceptibility. (A) Survival of SJ4005 and mutant animals on *E. coli* OP50 at 20°C. Day 0 represents young adults. *P* values for the values for mutant animals compared to the values for SJ4005 animals are as follows: *P* > 0.05 for *vit-1*(*ac2*), *P* < 0.001 for *vit-2*(*ac3*), *P* > 0.05 for *vit-4*(*ac4*), *P* < 0.001 for *vit-5*(*ac5*), and *P* > 0.05 for *vit-6*(*ac6*). (B) Survival of SJ4005 and mutant animals on *P. aeruginosa* PA14 at 20°C. The animals were grown on *E. coli* OP50 at 20°C until they were 1-day-old adults before being transferred to *P. aeruginosa* PA14. *P* values comparing the values for mutant animals relative to the values for SJ4005 animals are as follows: *P* < 0.01 for *vit-1*(*ac2*), *P* < 0.001 for *vit-2*(*ac3*), *P* < 0.001 for *vit-4*(*ac4*), *P* < 0.001 for *vit-5*(*ac5*), and *P* < 0.001 for *vit-6*(*ac6*). (C) Survival of SJ4005 and mutant animals on *E. coli* OP50 at 25°C. Day 0 represents young adults. *P* values comparing the values for mutant animals to the values for SJ4005 animals are as follows: *P* < 0.05 for *vit-1*(*ac2*), *P* < 0.001 for *vit-2*(*ac3*), *P* < 0.001 for *vit-4*(*ac4*), *P* < 0.001 for *vit-5*(*ac5*), and *P* < 0.001 for *vit-6*(*ac6*). Survival curves are representative assays of three independent experiments. *n* = 60 to 100. (D) Percent of animals alive after 5 days of adult life span at 20°C after *xbp-1* RNAi. The bar graph shows the means plus SD from three independent experiments.

There were no significant differences between the brood size of the mutants and that of SJ4005 animals (Fig. S6), suggesting that the accumulation in the intestine of a single VIT protein did not affect the overall transport of nutrients to oocytes. To examine the effects of the mutations on the life spans of the animals, we compared the survival of the mutants with that of SJ4005 animals grown on nonpathogenic *Escherichia coli* OP50. While *vit-1*(*ac2*), *vit-4*(*ac4*), and *vit-6*(*ac6*) did not show any significant differences in life span, *vit-2*(*ac3*) and *vit-5*(*ac5*) had a significantly reduced life span at 20°C (Fig. 5A and Table S1). In contrast, all of the mutants showed significantly enhanced susceptibility to the pathogenic bacteria *P. aeruginosa* PA14 at 20°C (Fig. 5B and Table S1). These results indicated that while some mutants might have been able to survive as long as SJ4005 animals under nonstressed conditions, under the stress caused by infection, all of the mutants exhibited reduced survival compared to the SJ4005 animals. Consistent with the idea that an increase in temperature enhances proteotoxic stress (28), all of the mutants displayed reduced survival when grown on *E. coli* OP50 at 25°C (Fig. 5C and Table S1). The mutant animals also showed enhanced susceptibility to another pathogen, *Salmonella enterica* (Fig. S7).

10.1128/mBio.00778-17.6 FIG S6  The brood size of the mutants does not change. The mean brood sizes of the animals at 20°C are shown. *P* values for all the mutants relative to SJ4005 animals are > 0.05. Download FIG S6, TIF file, 0.6 MB. Copyright © 2017 Singh and Aballay. 2017 Singh and Aballay This content is distributed under the terms of the Creative Commons Attribution 4.0 International license.

10.1128/mBio.00778-17.7 FIG S7  The mutants have enhanced susceptibility to the pathogenic bacteria *Salmonella enterica*. Survival of SJ4005 and mutant animals on *S. enterica* at 25°C is depicted. The animals were grown on *E. coli* OP50 at 20°C until they were 1-day-old adults before transferring the animals to *S. enterica* at 25°C. The *P* values for the mutant animals compared to the values for SJ4005 animals are as follows: *P* < 0.001 for *vit-1*(*ac2*), *P* < 0.0001 for *vit-4*(*ac4*), *P* < 0.0001 for *vit-5*(*ac5*), and *P* > 0.1 for *vit-6*(*ac6*). Download FIG S7, TIF file, 0.3 MB. Copyright © 2017 Singh and Aballay. 2017 Singh and Aballay This content is distributed under the terms of the Creative Commons Attribution 4.0 International license.

The mutant animals exhibited a reduced life span upon *xbp-1* RNAi, and more than 50% of the mutant animals died within 5 days of adulthood (Fig. 5D). Taken together, these results show that the increased ER stress caused by accumulation of VIT proteins reduces the life span and enhances the pathogen susceptibility of animals, indicating that the upregulated UPR helped to alleviate the toxic effects of the ER stress on the animals. The results also indicate that the enhanced susceptibility of *vit-1*(*ac2*), *vit-4*(*ac4*), and *vit-6*(*ac6*) animals to pathogen infection at 20°C is not due to general sickness, suggesting that mutations in VIT proteins reduce immunity against bacterial infections.

### High levels of VIT proteins enhance susceptibility to pathogen infection and contributes to immunosenescence.

**FIG 6:**
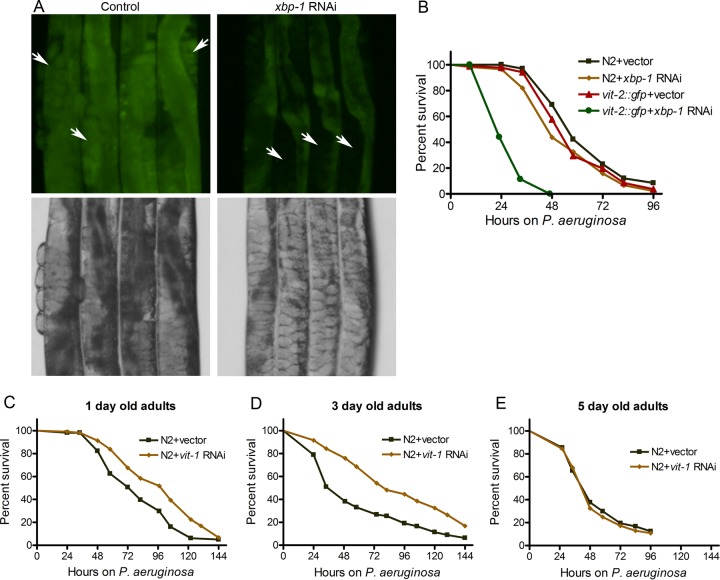
High levels of VIT proteins enhance susceptibility to pathogen infection. (A) Photomicrographs of 1-day-old adult transgenic animals expressing VIT-2::GFP with control and *xbp-1* RNAi. The white arrows point to embryos. (B) Survival of wild-type N2 animals and animals expressing VIT-2::GFP on *P. aeruginosa* PA14 at 25°C after treatment with control RNAi or *xbp-1* RNAi. The animals were grown on RNAi plates at 20°C until they were 1-day-old adults before being transferred to *P. aeruginosa* PA14 at 25°C. Comparing the value for control RNAi versus *xbp-1* RNAi for animals expressing VIT-2::GFP, *P* < 0.001. (C to E) Survival of N2 animals on *P. aeruginosa* PA14 at 25°C after treatment with control RNAi and *vit-1* RNAi. The animals were grown on RNAi plates at 20°C until they were 1-day-old, 3-day-old, and 5-day-old adults before being transferred to *P. aeruginosa* PA14 at 25°C.

It is known that the UPR decreases with aging (8, 9), with a concomitant increase in the levels of VIT proteins (29, 30). It is also known that the immune response decreases with aging (31, 32). To examine whether protein accumulation with aging may affect immunity, we decided to mimic some aspects of aging by blocking the UPR and by overexpressing VIT-2. While control RNAi animals displayed VIT-2::GFP mainly in the embryos, *xbp-1* RNAi animals primarily showed VIT-2::GFP in the intestine (Fig. 6A and Fig. S8). Consistent with our previous results indicating that VIT protein accumulation affects intestinal physiology, animals expressing VIT-2::GFP exhibited pale intestines upon *xbp-1* RNAi but not control RNAi (Fig. 6A and Fig. S8). As shown in Fig. 6B, *xbp-1* RNAi of animals expressing VIT-2::GFP had an enhanced susceptibility to *P. aeruginosa* infection compared to control RNAi. These results suggest that the accumulation of VIT protein in the intestine may negatively affect the response to infection. To address whether accumulation of VIT proteins with age affects the response to infection, we studied the pathogen susceptibility of aging animals upon knockdown of *vit* genes. We found that RNAi against *vit-1*, which is known to inhibit expression of other *vit* genes (30), enhanced the resistance to *P. aeruginosa* infection of 1-day-old adult animals (Fig. 6C). The resistance to *P. aeruginosa* infection upon *vit-1* RNAi was further enhanced in 3-day-old adult animals (Fig. 6D). Aging *C. elegans* nematodes are known to have diminished UPR (9) and enhanced aggregation of the proteome (33, 34), and this overall proteotoxic stress correlates with enhanced susceptibility to pathogen infection (comparison of Fig. 6C to E). As shown in Fig. 6C and D, *vit-1* RNAi, which reduced the expression of one of the most abundant intestinal proteins, improved the resistance to *P. aeruginosa* infection of mildly aged *C. elegans*. However, *vit-1* RNAi had no effect on the resistance to *P. aeruginosa* infection in 5-day-old adult animals (Fig. 6E), suggesting that the deleterious effects of the overall misfolded proteome of 5-day-old animals cannot be reversed by only inhibiting VIT protein expression. Taken together, these results suggest that the accumulation of VIT proteins in the intestine may affect not only longevity but also immunity and that they may contribute to immunosenescence.

10.1128/mBio.00778-17.8 FIG S8  Blockage of the UPR gene *xbp-1* leads to intestinal accumulation of VIT proteins. Photomicrographs of 1-day-old adult transgenic animals expressing VIT-2::GFP with control and *xbp-1* RNAi. Download FIG S8, TIF file, 1 MB. Copyright © 2017 Singh and Aballay. 2017 Singh and Aballay This content is distributed under the terms of the Creative Commons Attribution 4.0 International license.

### VIT accumulation leads to reduced gene expression of genes encoding secreted immune effectors.

**FIG 7:**
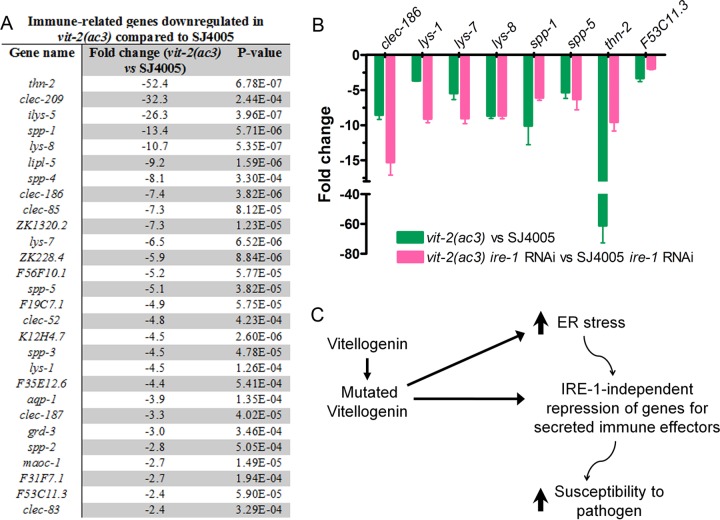
*vit-2*(*ac3*) animals exhibit reduced expression of immune genes. (A) List of immune-related genes that are downregulated in *vit-2*(*ac3*) animals compared to SJ4005 animals. (B) qRT-PCR for selected immune genes. The bar graph shows the means plus SD for three independent experiments. Comparing the values of all genes in *vit-2*(*ac3*) animals versus SJ4005 animals, *P* < 0.0001. (C) Model for the effects of mutations in vitellogenin proteins on innate immunity. Mutations in vitellogenin proteins lead to enhanced ER stress. Accumulation of mutated vitellogenin proteins and/or enhanced ER stress lead to suppression of secreted immune effectors in an IRE-1-independent manner, resulting in an enhanced susceptibility to pathogen infection.

To understand the mechanism of enhanced susceptibility to pathogen infection caused by VIT accumulation, we used RNA sequencing to focus on transcriptional changes induced by VIT protein accumulation in *vit-2*(*ac3*) animals compared to SJ4005 animals. Of the 542 differentially regulated genes, 186 genes were upregulated, while 356 genes were downregulated (Table S2). Consistent with the idea that VIT protein accumulation induces ER stress, gene ontology analysis for biological function of the upregulated genes showed enrichment in genes linked to ER stress and the UPR (Table S2). Interestingly, we found that a subset of downregulated genes was enriched in ontology clusters associated with defense response, innate immune response, and defense responses to Gram-negative and Gram-positive bacteria (Table S2). These downregulated genes included lysozymes, saposin-like proteins (*spp* genes), and C-type lectins (Fig. 7A), which are conserved markers of immune activation that encode immune effectors secreted by the intestinal cells. For further analysis, we selected genes that have been experimentally linked to host defense against infections and confirmed their downregulation by quantitative reverse transcription-PCR (qRT-PCR) (Fig. 7B).

10.1128/mBio.00778-17.10 TABLE S2  List of differentially expressed genes in *vit-2*(*ac3*) versus SJ4005 and their gene ontology (GO) enrichment analysis Download TABLE S2, XLSX file, 0.1 MB. Copyright © 2017 Singh and Aballay. 2017 Singh and Aballay This content is distributed under the terms of the Creative Commons Attribution 4.0 International license.

Because enhanced ER stress is known to activate regulated IRE-1-dependent degradation (RIDD) of mRNAs (35), we studied whether genes encoding secreted immune effectors were repressed by IRE-1. Thus, we compared the mRNA levels of immune genes of *vit-2*(*ac3*) *ire-1* RNAi animals to those of SJ4005 *ire-1* RNAi animals. We found that the expression level of 7 out of 8 immune genes was significantly inhibited in *vit-2*(*ac3*) animals in an IRE-1-independent manner (Fig. 7B). Thus, only a single studied gene, *thn-2*, showed partial IRE-1-dependent inhibition. These studies indicate that the expression of immune genes encoding secreted proteins is downregulated by the ER stress caused by accumulation of VIT proteins in an IRE-1-independent manner.

## DISCUSSION

We demonstrated that mutations in lipoproteins in *C. elegans* led to their accumulation in the intestine and resulted in upregulation of the UPR. Although protein accumulation enhanced the UPR, which mitigated the adverse effects of accumulated VIT proteins on the isolated mutant animals, the animals exhibited enhanced susceptibility to pathogen infection and a shortened life span. Our results also indicate that the enhanced susceptibility to pathogen infection of animals exhibiting high ER stress due to accumulation of VIT proteins may be due to a mechanism activated by the resultant ER stress that inhibits the expression of a small subset of genes in the *C. elegans* genome that is highly enriched in immune effectors (Fig. 7C).

Surprisingly, even after cessation of the self-fertile reproductive period, the levels of VIT proteins continue to increase (29, 30, 36, 37). Moreover, not only does the amount of VIT proteins increase with age but also the rate of VIT protein synthesis increases (37). It is not clear why the VIT proteins are expressed even after cessation of the self-fertile reproductive period. One possibility is that VIT proteins remain necessary for reproduction by cross-fertilization, which occurs up to day 13 of adulthood (38) and is more than two times longer than the self-fertilization period. Another possibility is that the animals are not under selection pressure after the reproductive period, and hence, no active mechanism has evolved to inhibit VIT protein expression during this period.

Gene expression and proteomic studies of several long-lived mutants have shown that these animals have reduced levels of *vit* genes and VIT proteins (30, 39–42). In addition, RNAi against different *vit* genes prolongs life span in multiple studies (30, 40, 43). Interestingly, long-lived mutants, which have reduced levels of *vit* genes and VIT proteins, carry mutations affecting different processes that are capable of controlling longevity, such as the *daf-2*/*daf-16* pathway, sterility, dietary restriction, and AMP-activated protein kinase pathway, among others. Even chemical substances such as garlic extract (44) and tyrosol (45), which enhance life span, lead to reduced levels of VIT proteins in animals. VIT proteins were also found to be highly abundant in insoluble fractions of the proteomes from aged animals (33, 34), likely because of their aggregation. Because all of the different longevity pathways seem to merge at reduced levels of VIT proteins, these studies highlight that one of the most downstream strategies utilized to enhance life span could be a reduction of the expression of aggregation-prone proteins such as lipoproteins. It has been shown that animals with a longer life span would also possess improved pathogen resistance (46). This could be due, at least in part, to reduced levels of aggregation-prone proteins, such as lipoproteins. Indeed, we observed that *vit-1* RNAi enhanced the resistance of animals to pathogens.

Several bacterial pathogens are known to induce ER stress in host cells. In *C. elegans*, both Gram-positive and Gram-negative pathogenic bacteria have been shown to enhance protein aggregation (12). These findings are further supported by the observation that pathogenic *P. aeruginosa* bacteria upregulate the UPR (14). Moreover, *P. aeruginosa* infection led to reduced expression levels of genes *lys-7*, *spp-1*, and *thn-2*, which are required for optimal survival on *P. aeruginosa* (47). We found that these genes, which are also markers of immune activation by exposure to other pathogenic bacteria such as *Salmonella enterica* serovar Typhimurium and *Enterococcus faecalis*, were downregulated by the ER stress caused by VIT accumulation. Taken together, these results suggest that the ER stress caused by *P. aeruginosa* infection leads to downregulation of genes encoding secreted immune effectors.

In summary, our results provide evidence that the ER stress caused by protein accumulation results in the downregulation of genes encoding secreted proteins. Enhanced ER stress is known to lead to repression of specific mRNAs, which encode components of the plasma membrane and secreted proteins, by their degradation by IRE-1 (35). Here, we show that suppression of the immune genes encoding secreted proteins is independent of IRE-1. Our results also provide a mechanistic explanation for the adverse effects that high ER stress has on immunity. In future studies, it will be important to characterize the IRE-1-independent pathway(s) that may sense increased ER stress and elicit the suppression of genes encoding secreted proteins.

## MATERIALS AND METHODS

### Bacterial strains.

The following bacterial strains were used: *Escherichia coli* OP50, *E. coli* HT115(DE3), *Pseudomonas aeruginosa* PA14, and *Salmonella enterica* serovar Typhimurium 1344. The cultures for these bacteria were grown in Luria-Bertani (LB) broth at 37°C.

### *C. elegans* strains and growth conditions.

The *C. elegans* parental strain SJ4005 (*Phsp-4*::*gfp*) and wild-type Bristol N2 strains were obtained from the Caenorhabditis Genetics Center (University of Minnesota, Minneapolis, MN). The *C. elegans* strains were cultured under standard conditions and fed *E. coli* OP50.

### Forward genetic screen for UPR upregulation.

Ethyl methanesulfonate (EMS) mutagenesis was performed using the SJ4005 strain, which expresses green fluorescent protein (GFP) under the promoter of the *hsp-4* gene. Approximately 3,000 synchronized late L4 larvae of SJ4005 were treated with 50 mM EMS for 4 h and then washed three times with M9 medium. The washed animals (P0 generation) were then transferred to four large petri dishes (15 cm) containing *E. coli* OP50 and allowed to lay eggs of F_1_ progeny overnight. The P0 animals were then washed away with M9 medium, while the F_1_ eggs remained attached to the bacterial lawn. The F_1_ eggs were allowed to grow to adulthood. The animals that showed enhanced GFP expression in the F_1_ progeny were selected on individual plates. Approximately 80,000 haploid genomes were mutated in the screen. All of the mutants were backcrossed six times with the parental SJ4005 strain before analysis.

### Fluorescence imaging.

Animals were anesthetized using an M9 salt solution containing 30 mM sodium azide and mounted onto 2% agar pads. The animals were then visualized using a Leica M165 FC fluorescence stereomicroscope.

### RNA interference.

RNA interference (RNAi) was used to generate loss-of-function RNAi phenotypes by feeding nematodes *E. coli* strain HT115(DE3) expressing double-stranded RNA (dsRNA) homologous to a target gene (48, 49). Briefly, *E. coli* with the appropriate vectors were grown in LB broth containing ampicillin (100 μg/ml) and tetracycline (12.5 μg/ml) at 37°C overnight and plated onto NGM plates containing 100 μg/ml ampicillin and 3 mM isopropyl-β-d-thiogalactoside (IPTG) (RNAi plates). RNAi-expressing bacteria were allowed to grow overnight at 37°C. Gravid adults were transferred to RNAi-expressing bacterial lawns and allowed to lay eggs for 2 h. The gravid adults were removed, and the eggs were allowed to develop at 20°C to 1-day-old adults (96 h at 20°C from eggs) for subsequent assays. For age-dependent assays of N2 on *vit-1* RNAi, the eggs were allowed to reach 1-day-old adult stage on *vit-1* RNAi plates at 20°C (96 h at 20°C from eggs), 3-day-old adult stage (144 h at 20°C from eggs), and 5-day-old adult stage (192 h at 20°C from eggs) before transferring them to *P. aeruginosa* PA14 plates. *unc-22* RNAi was included as a positive control in all experiments to account for the RNAi efficiency. RNAi clones for *vit-2* and *vit-6* are unavailable in the libraries of J. Ahringer and M. Vidal. The rescue of the enhanced UPR in *vit-2*(*ac3*) was tested by *vit-1* RNAi thanks to cross-reactivity. The rescue of the enhanced UPR in *vit-6*(*ac6*) was not tested by RNAi due to the lack of cross-reactivity.

### Whole-genome sequencing and data analysis.

The mutant animals were grown at 20°C on NGM plates seeded with *E. coli* OP50 until starvation. The animals were rinsed off the plates with M9 medium, washed three times, incubated in M9 medium with rotation for 2 h to eliminate food from the intestine, and washed again three times with M9 medium. Genomic DNA extraction was performed using the Gentra Puregene kit (Qiagen, Netherlands). DNA libraries were prepared according to a standard Illumina (San Diego, CA) protocol. The DNA was subjected to whole-genome sequencing (WGS) on an Illumina HiSeq 4000 sequencing platform using 50 single-end nucleotide reads. Library preparation and WGS were performed at the Duke Center for Genomic and Computational Biology.

The whole-genome sequence data were analyzed using the EMS density mapping workflow from the CloudMap program of the Galaxy web platform (50). The list of single nucleotide polymorphisms (SNPs) was generated by comparison to the reference genome WS220. Common SNPs were subtracted, and linkage maps were generated.

### RNA isolation and quantitative reverse transcription-PCR.

Animals were synchronized by egg laying. One hundred gravid adult animals were transferred to a 10-cm plate seeded with *E. coli* OP50 and allowed to lay eggs for 4 to 5 h. The gravid adults were then removed, and the eggs were allowed to develop at 20°C for 96 h. The animals were then collected, washed with M9 buffer, and frozen in TRIzol reagent (Life Technologies, Inc., Carlsbad, CA). Total RNA was extracted using the RNeasy Plus Universal kit (Qiagen, Netherlands). Residual genomic DNA was removed using TURBO DNase (Life Technologies, Inc., Carlsbad, CA). A total of 6 μg of total RNA was reverse transcribed with random primers using the High-Capacity cDNA reverse transcription kit (Applied Biosystems, Foster City, CA).

Quantitative reverse transcription-PCR (qRT-PCR) was conducted using the Applied Biosystems one-step real-time PCR protocol using SYBR green fluorescence (Applied Biosystems) on an Applied Biosystems 7900HT real-time PCR machine in 96-well plate format. Twenty-five-microliter reaction mixtures were analyzed as outlined by the manufacturer (Applied Biosystems). The relative fold changes of the transcripts were calculated using the comparative cycle threshold (*C*_*T*_) (2^−ΔΔ*CT*^) method and normalized to pan-actin (*act-1*, *act-3*, and *act-4*). The cycle thresholds of the amplification were determined using StepOnePlus software (Applied Biosystems). All samples were run in triplicate. The primer sequences are available upon request.

### RNA sequencing and data analysis.

Total RNA samples for SJ4005 and *vit-2*(*ac3*) animals were isolated as described above. Three biological replicates were used to prepare RNA libraries using a Kappa standard mRNA sequencing kit. The RNA was sequenced on an Illumina HiSeq 4000 sequencing platform using 50 single-end nucleotide reads. Library preparation and sequencing were performed at the Duke Center for Genomic and Computational Biology.

The RNA sequence data were analyzed using Partek. Briefly, the RNA reads were aligned to the *C. elegans* genome (WS255) using the aligner STAR. Counts were normalized for sequencing depth and RNA composition across all samples. Differential gene expression analysis was then performed on normalized samples. Genes exhibiting at least twofold change and a false-discovery rate (FDR) of 1% or less were considered differentially expressed. Gene ontology analysis was performed using the DAVID Bioinformatics Database (david.abcc.ncifcrf.gov/).

### Fatty acid supplementation assay.

Fatty acid supplementation plates were made using an adapted protocol (51). Briefly, fresh 100 mM aqueous solution stocks of each supplement were made and added to NGM medium that had been cooled to 55°C to obtain a final concentration of 300 µM. The fatty acid salts were purchased from Sigma.

### Generation of *C. elegans* transgenic lines.

The plasmid V2B3, which encodes a functional VIT-2(YP170B)::GFP fusion protein expressed under the *vit-2* promoter, was a generous gift from Barth Grant (19). Site-directed mutagenesis was used to generate *vit-2*(*G839R*)::*gfp* plasmid. N2 wild-type animals were microinjected with *vit-2*::*gfp* plasmids along with pCFJ90 (*Pmyo-2*::*mCherry*) as a coinjection marker. The *vit-2*::*gfp* plasmids were used at a concentration of 25 ng/µl, while the coinjection marker was used at a concentration of 5 ng/µl. The plasmids were maintained as extrachromosomal arrays, and at least two independent lines were maintained for each plasmid.

### Brood size assay.

Five L4 animals were transferred to individual plates and incubated at 20°C. The animals were transferred to fresh plates every other day, and progeny were counted and removed every day.

### *C. elegans* longevity assays.

Life span assays were performed on NGM plates containing *E. coli* OP50 or RNAi plates containing *E. coli* HT115(DE3) with the appropriate vector. Animals were scored as alive, dead, or gone every day. Animals that failed to display touch-provoked movement were scored as dead. Experimental groups contained 60 to 100 animals. The prefertile period of adulthood was used as *t* = 0 for the life span analysis. The assays were performed at 20 and 25°C.

### *C. elegans* killing assays on *Pseudomonas aeruginosa* PA14.

The bacterial lawns used for *C. elegans* killing assays were prepared by spreading 20 µl of an overnight culture grown at 37°C of *P. aeruginosa* PA14 on the complete surface of modified NGM agar medium (0.35% peptone instead of 0.25% peptone) in 3.5-cm-diameter plates. The plates were incubated at 37°C for 12 to 16 h and then cooled to room temperature for at least 1 h before seeding with synchronized 1-day-old adult animals. The killing assays were performed either at 20°C or at 25°C as mentioned, and live animals were transferred daily to fresh plates. Animals were scored at the times indicated and were considered dead when they failed to respond to touch.

### *C. elegans* killing assays on *Salmonella enterica*.

The bacterial lawns used for *C. elegans* killing assays were prepared by placing 20 µl of an overnight culture grown at 37°C of *S. enterica* on modified NGM agar medium (0.35% peptone instead of 0.25% peptone) in 3.5-cm-diameter plates. The plates were incubated at 37°C for 12 to 16 h and then cooled to room temperature for at least 1 h before seeding with synchronized 1-day-old adult animals. The killing assays were performed at 25°C, and live animals were transferred daily to fresh plates. Animals were scored at the times indicated and were considered dead when they failed to respond to touch.
