# Supplementary figures and images for: Endoplasmic Reticulum Stress Caused by Lipoprotein Accumulation Suppresses Immunity against Bacterial Pathogens and Contributes to Immunosenescence
Source: mBio. 2017 May 30;8(3):e00778-17. doi: 10.1128/mBio.00778-17 (PMC5449662; doi:10.1128/mBio.00778-17)

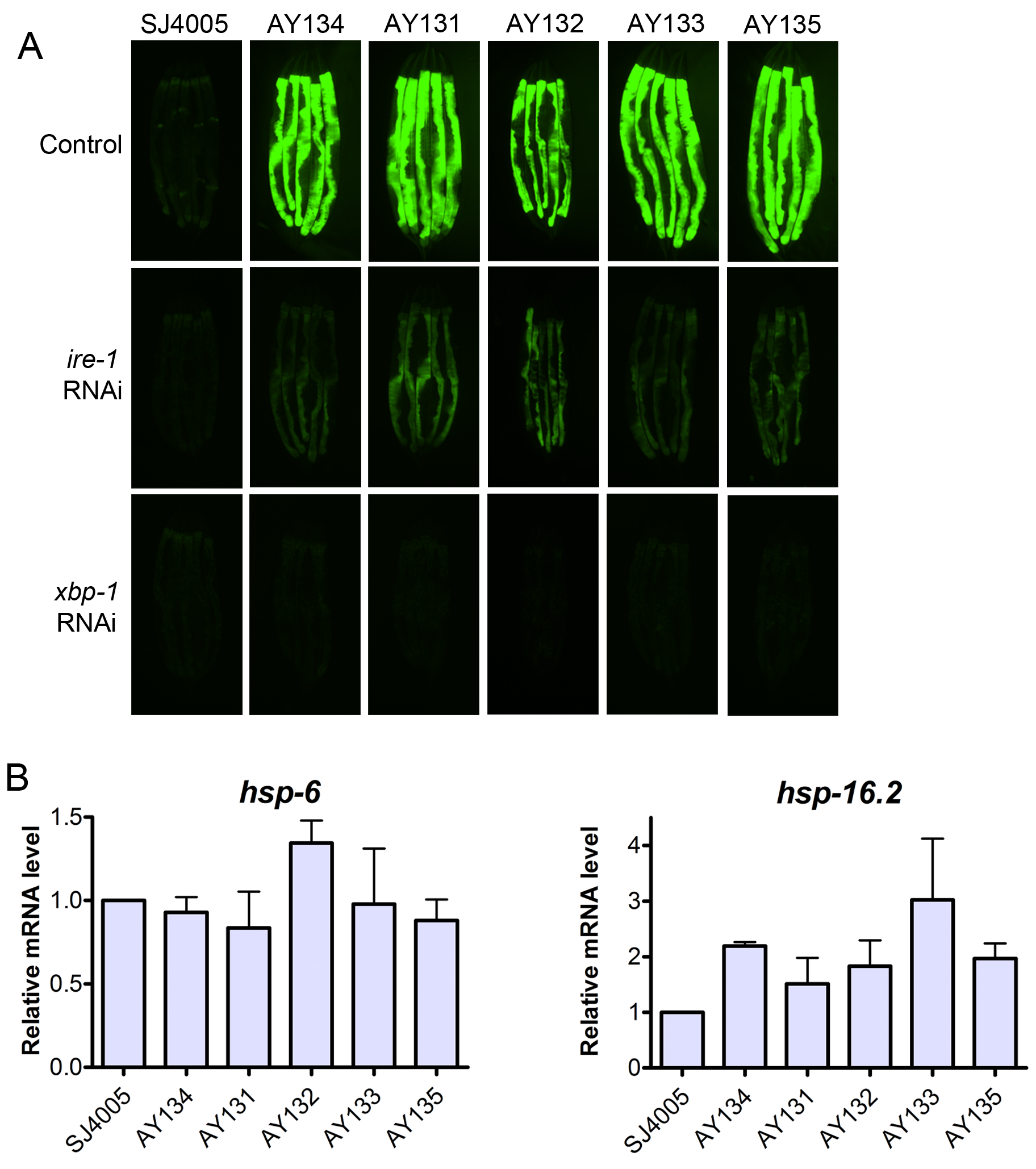

Supplement: FIG S1 [file mbo003173330sf1.tif]

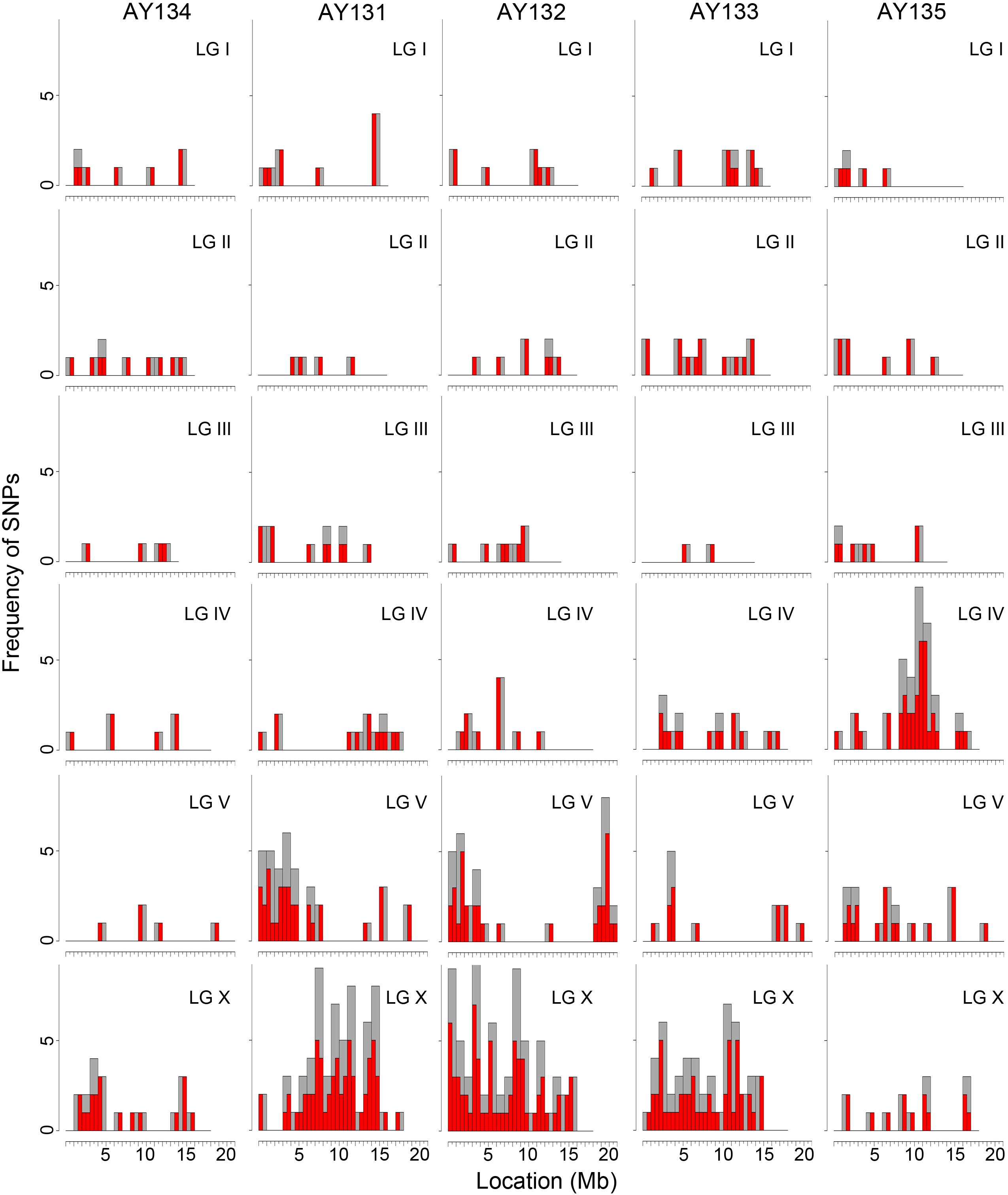

Supplement: FIG S2 [file mbo003173330sf2.tif]

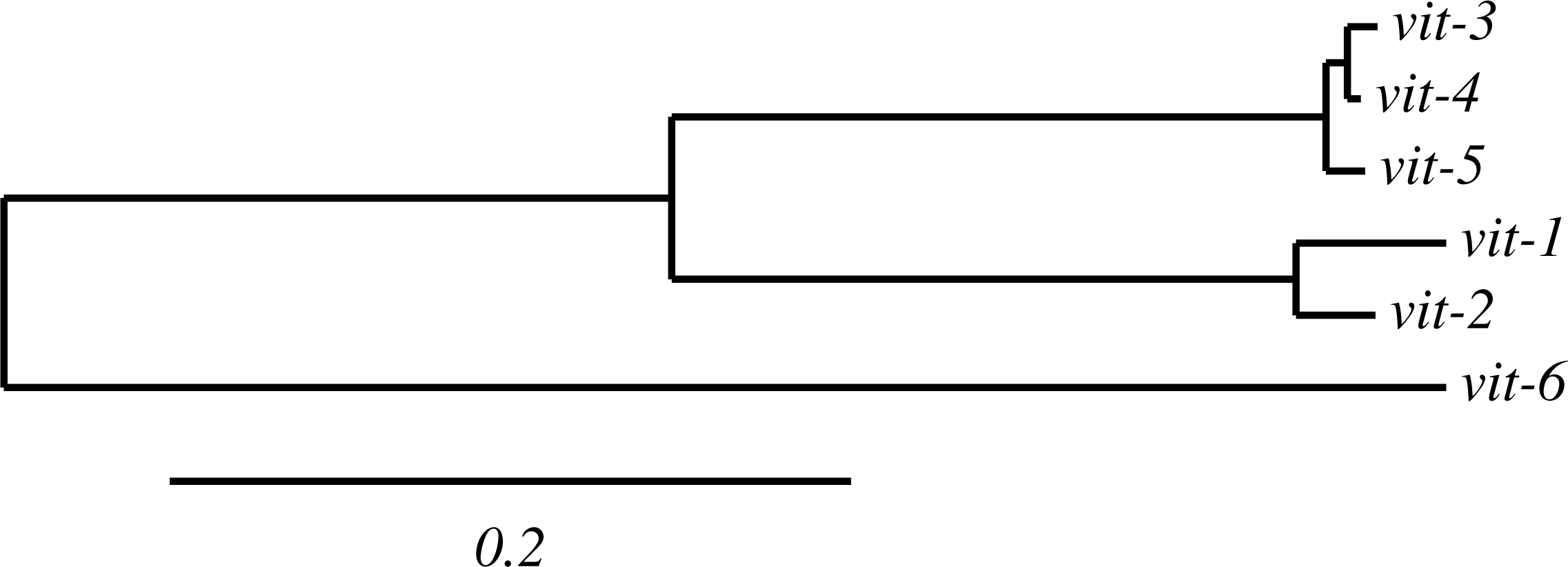

Supplement: FIG S3 [file mbo003173330sf3.tif]

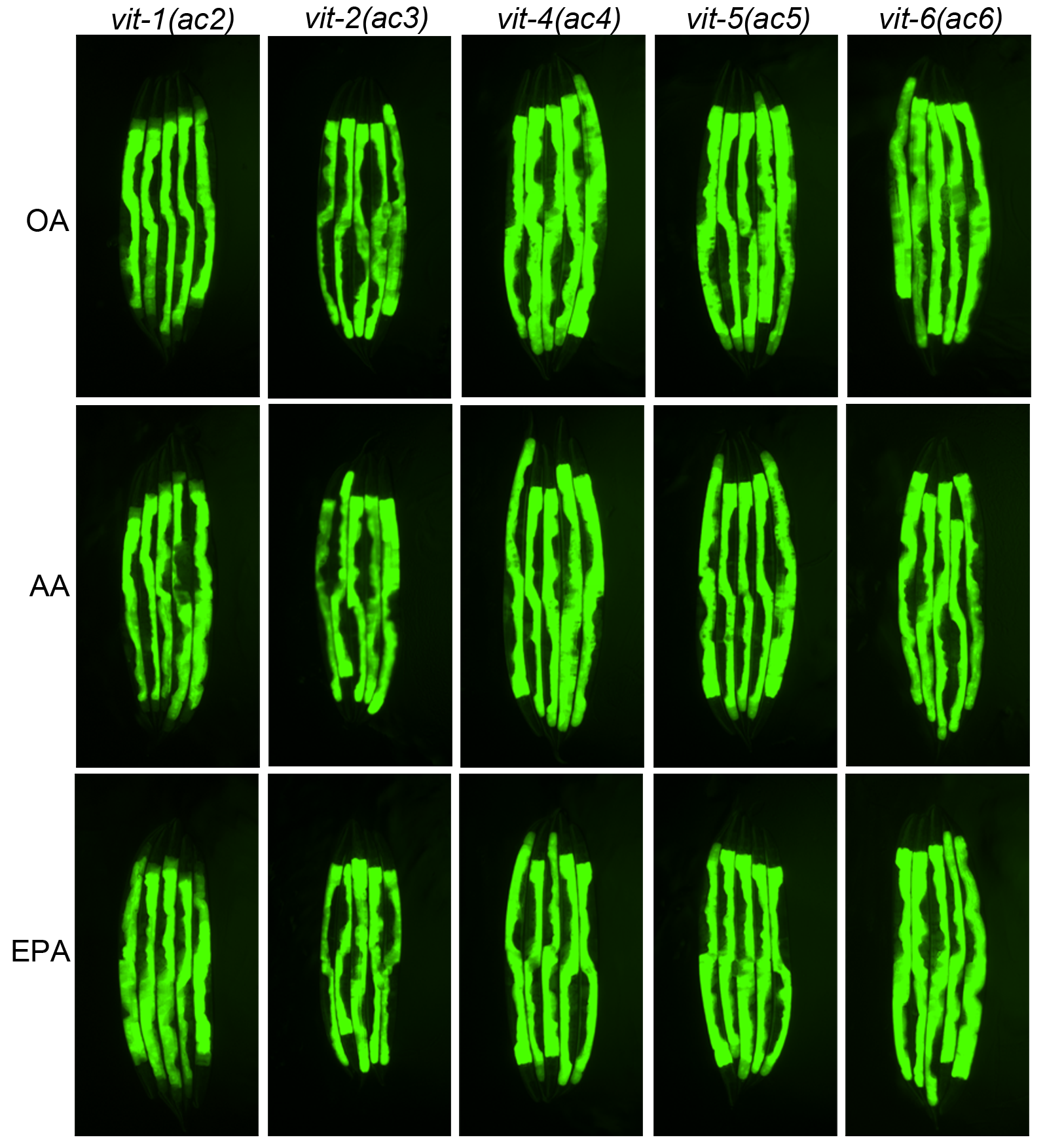

Supplement: FIG S4 [file mbo003173330sf4.tif]

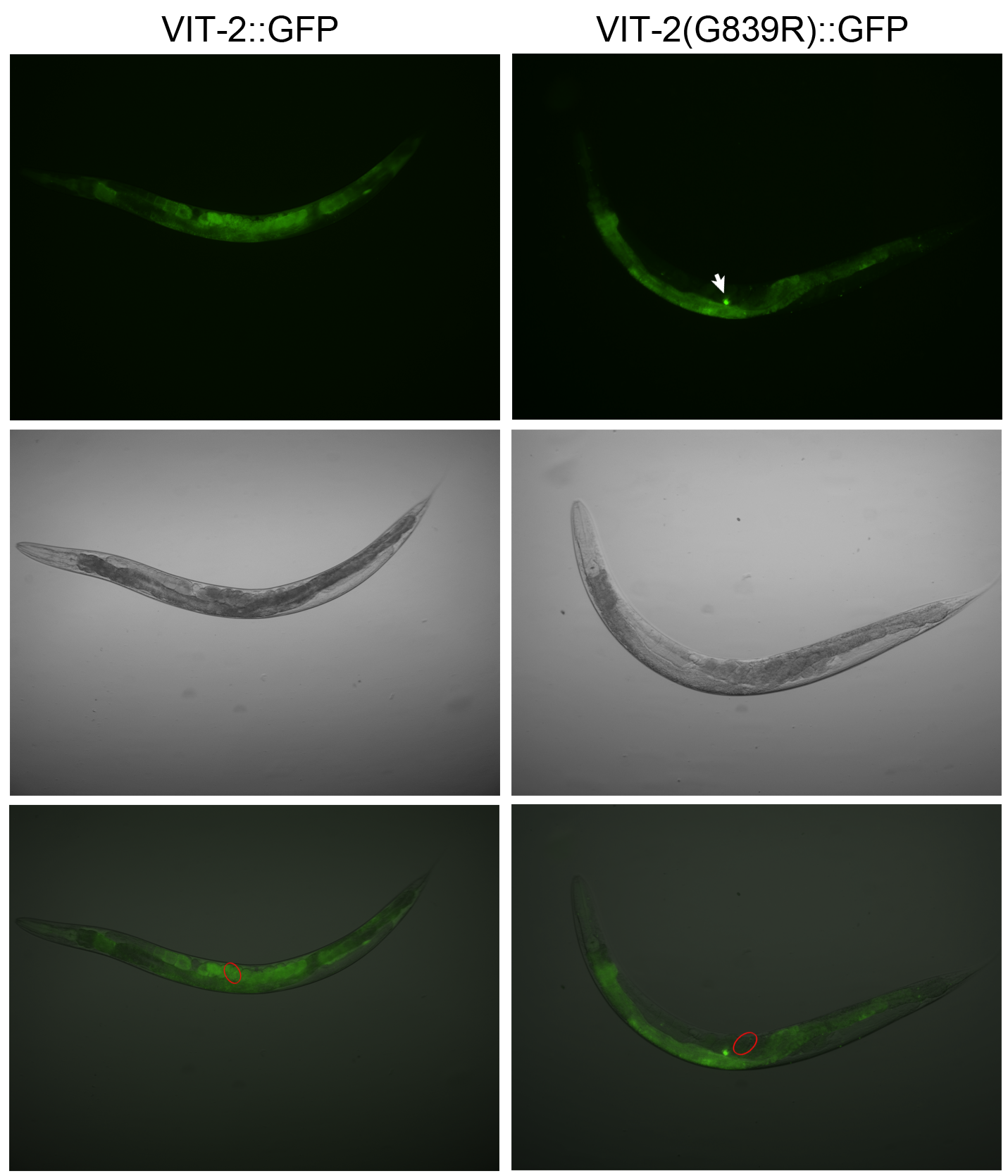

Supplement: FIG S5 [file mbo003173330sf5.tif]

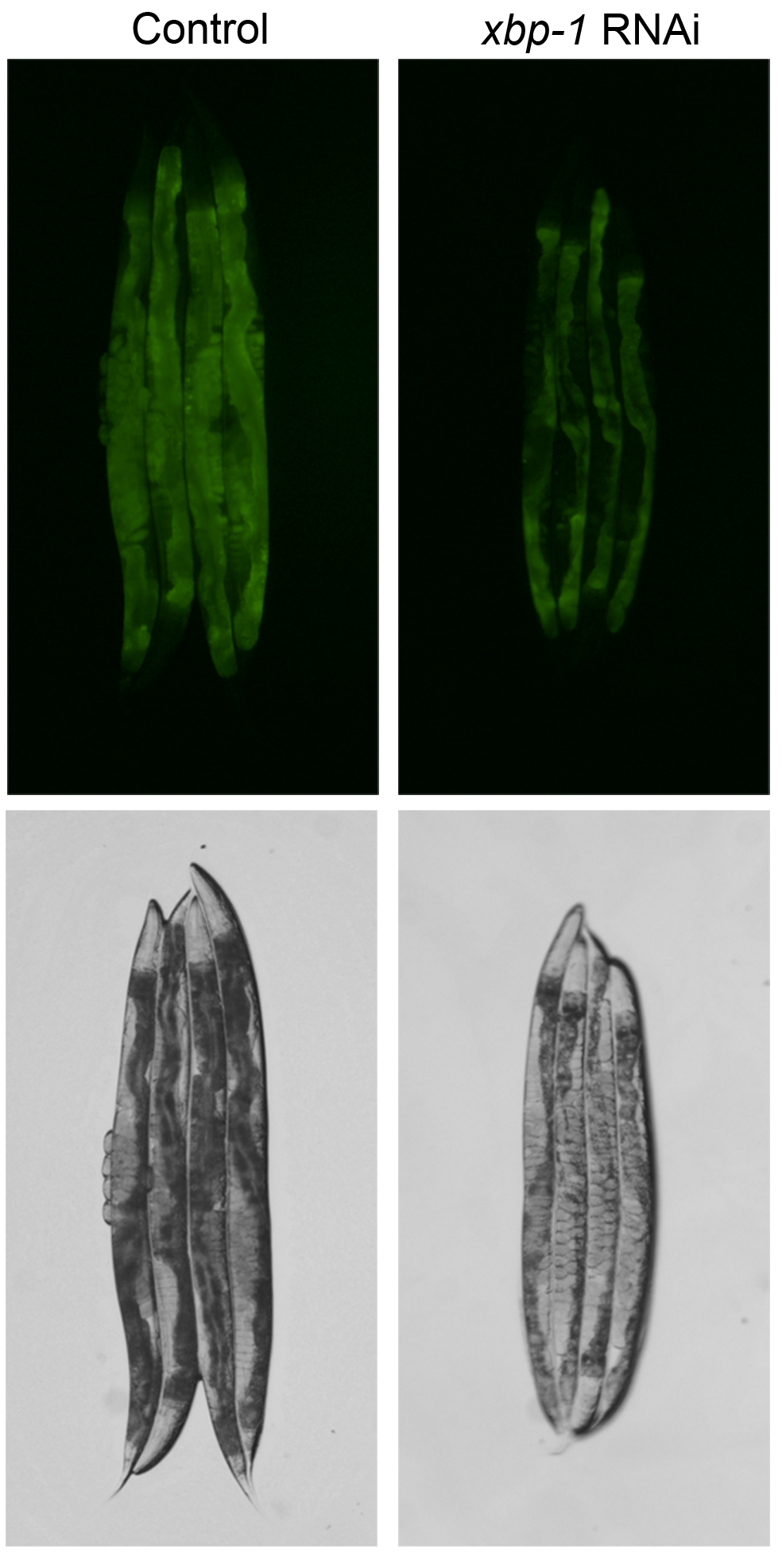

Supplement: FIG S8 [file mbo003173330sf8.tif]
